# Auditing causality of the multiple-burden of malnutrition in India and South Africa: a critical need for directions

**DOI:** 10.1017/S1368980023001921

**Published:** 2023-12

**Authors:** Angeline Jeyakumar, Hema Kesa, Swapnil Godbharle

**Affiliations:** 1 Department of Nutrition, University of Nevada, Reno, Nevada, USA; 2 Food Evolution Research Laboratory (FERL), School of Tourism and Hospitality, College of Business and Economics, University of Johannesburg, Auckland Park, 2006, South Africa; 3 Department of Health Sciences, Savitribai Phule Pune University, Pune 411007, MH, India

**Keywords:** Dietary patterns, Nutrition transition, India, South Africa

## Abstract

Developing economies are shaped by the current predicament of urbanisation and its impact on health is inevitable. In the post-pandemic times, India and South Africa witnessed a GDP growth rate of about 1·7 % and 1·9 %, respectively, while the developed economies like Europe and the USA have bounced back with more than 2 % GDP. The similarities and differences between India and South Africa provide potential candidates to study nutrition transition with the elements of urbanisation. In both countries, increased access to convenience foods is a consequence of the rapid expansion of small and medium enterprises, open international markets and expanding food supply chains. Also, there has been significant acculturation and people have moved away from traditional diets in these two countries. A spate of similar changes in the food environment is a telling sign of serious ill-health consequences in both countries. Generating evidence on causality is fundamental to informing policy. India and South Africa qualify as potential candidates to study the multiple burdens of malnutrition. Collaborating with different disciplines such as data sciences and capacitating analytic skills are key to progress in this direction.

Urbanisation is ‘the process by which towns and cities are formed and become larger as more and more people begin living and working in central areas’^(1)^. The concept of urbanisation rested on the desire to provide more opportunities, prevent pollution and create a community for potential growth in the future^(2)^. However, the term gradually lost its implications leading to the birth of modern urbanised societies. The World Bank recorded that the percentage increase in the urban population accounted from 52 % in 2011 to 56·5 % in 2021^(3)^. The seeming success of swift urbanisation driven by technology has resulted in significant inequalities, poverty, environmental pollution and health hazards^(4)^.

## Changing demography

Developing economies are shaped by the current predicament of urbanisation and its impact on health is inevitable. Urbanisation has featured transitions in socio-economic, demographic shifts and beyond. In the post-pandemic times, India and South Africa witnessed a GDP growth rate of about 1·7 % and 1·9 %, respectively^(5,6)^, while the developed economies like Europe and the USA have bounced back with more than 2 % GDP^(7)^. The population in India to date is close to 1·5 billion, while that of South Africa is over 60 million^(8,9)^. The population in Europe is over 700 million and in the USA over 300 million. The mean age of Indians and South Africans is 28·7 and 27·6 years, respectively, while that of Europe and the USA is above 30 years^(5,6)^. The similarities and differences between India and South Africa provide potential candidates to study nutrition transition with the elements of urbanisation. Younger demography is suggestive of increased health and reproductive needs, and adequate income to sustain health. The nutritional needs of the young in growing economies are deeply relevant considering the intergenerational perpetuation of malnutrition. Poor nutrition imposes significant economic decline compounded with less productivity^(10,11)^.

## Changing food environment and dietary choices

A particular challenge of urbanisation in growing economies, characterised by young demography, rests in dietary choices. Food consumption behaviours reflect less preparation of food at home and increased consumption of food away from home, combined with a greater preference for fast foods, ready-to-eat takeaways and processed and ultra-processed foods. The transition is obvious in national data sets in India, where the misconception of the declining calorie consumption often cited as the ‘consumption puzzle’ was the result of a missed calculation of the energy consumed away from home^(12)^. India has seen a substantial increase in food away from home from 23 % in 1994 to 45 % in 2011–2012^(13)^. Evidence from South Africa is nonetheless similar to India, recognisable from the growth of the food away from home sector^(14)^. Choices of fast foods contribute to an increased intake of dietary fats compared with food away from home in South Africa^(15)^. In both countries, increased access to convenience foods is a consequence of the rapid expansion of small and medium enterprises, open international markets and expanding food supply chains^(16,17)^. Better incomes and smaller families improve the affordability of convenience foods. The higher concern is the penetration of food outlets in residential areas, and the convenience of online purchases is typical characteristics of urbanisation, characterised by less time for food preparation and women contributing to the family’s income^(18)^. In both countries, there has been significant acculturation and people have moved away from traditional diets^(19–21)^. A spate of similar changes in the food environment is a telling sign of serious ill-health consequences in both countries.

## Changing and unchanging statistics

Changing dietary patterns and their association with increased human health risks such as obesity and hypertension, and non-communicable diseases such as type 2 diabetes mellitus, heart disease and stroke are well established. The national surveys in India and Africa reflect a steady increase in the prevalence of overweight and obesity among adults, a high prevalence of non-communicable diseases and negligible reductions in the prevalence of undernutrition and micronutrient deficiencies. Close to a 3% increase in overweight and obesity, over 5 years in India, the association between wealth index and overnutrition is quite evident. However, the burden of undernutrition and micronutrient deficiencies is quite unrelenting^(22–24)^.

## Questions unanswered

The present trends leave many epidemiological questions unanswered. Is the evidence of types and trends of multiple burdens of malnutrition substantial? Have we characterised the double or multiple burdens of malnutrition in the context of varying economies, cultures or demographics? Do countries with similarities show similar trends in non-communicable diseases? Are the Indian and South African health systems resilient to address the unknown multiple burdens? How reliable and generalisable is the evidence from cross-sectional and observational studies? In countries with fragile public health systems do interventions improve the quality of life? Do observational and cross-sectional studies provide enough evidence of causality?

## Way forward

Generating evidence on causality is fundamental to informing policy. In the era of greater national surveillance and data generation, the need for meta-research has emerged. The limitations and gaps could be identified and addressed by ushering in new analyses and comparisons. India and South Africa qualify as potential candidates to study the multiple burdens of malnutrition. Collaborating with different disciplines such as data sciences and capacitating analytic skills are key to progress in this direction. A critical need for these countries is to investigate the causality of malnutrition and shift in dietary practices from traditional foods using nationally representative data. This will enable policy decisions to prevent the malnutrition burden keeping traditional foods as the core.
